# MEGA-V: detection of variant gene sets in patient cohorts

**DOI:** 10.1093/bioinformatics/btw809

**Published:** 2016-12-21

**Authors:** Gennaro Gambardella, Matteo Cereda, Lorena Benedetti, Francesca D Ciccarelli

**Affiliations:** Division of Cancer Studies, King’s College London, London, UK

## Abstract

**Summary:**

Detecting significant associations between genetic variants and disease may prove particularly challenging when the variants are rare in the population and/or act together with other variants to cause the disease. We have developed a statistical framework named Mutation Enrichment Gene set Analysis of Variants (MEGA-V) that specifically detects the enrichments of genetic alterations within a process in a cohort of interest. By focusing on the mutations of several genes contributing to the same function rather than on those affecting a single gene, MEGA-V increases the power to detect statistically significant associations.

**Availability and Implementation:**

MEGA-V is available at https://github.com/ciccalab/MEGA

**Supplementary information:**

Supplementary data are available at *Bioinformatics* online.

## 1 Introduction

Despite the large amount of data from genome-wide association studies, still a considerable fraction of genetic diseases lacks significant associations with causative variants. Different reasons account for the missing heritability, including the disease-causative role of rare variants, the cumulative effect of multiple variants to the disease phenotype, and/or the alterations of different genes perturbing the same biological process. In all these cases the detection of significant associations is challenging because commonly used approaches lack statistical power. To solve this, some methods collapse variants within a genomic region thus increasing the overall signal (Lee *et al.*, 2014; Pan *et al.*, 2014; Wu *et al.*, 2011). However, these methods are designed to detect associations of few variants or single genes but not of multiple genes or biological functions. Here, we present MEGA-V (Mutation Enrichment Gene set Analysis of Variants) a statistical framework to identify biological processes that are significantly over mutated in specific cohort of patients. MEGA-V systematically aggregates genetic variants into pre-defined gene sets and then identifies those gene sets with significant over-representations of variants in the cohort of interest. The founding principle of MEGA-V is similar to that of gene set analysis (GSA) used to identify functionally related genes that show significant differences between biological states or phenotypes (de Leeuw *et al.*, 2016; Subramanian *et al.*, 2005). Starting from the mutation counts in a cohort of samples, MEGA-V applies a GSA-like approach to detect significantly altered processes, without any prior additional measure of association between the variants and the phenotype of interest.

## 2 Methods

**Fig. 1.**
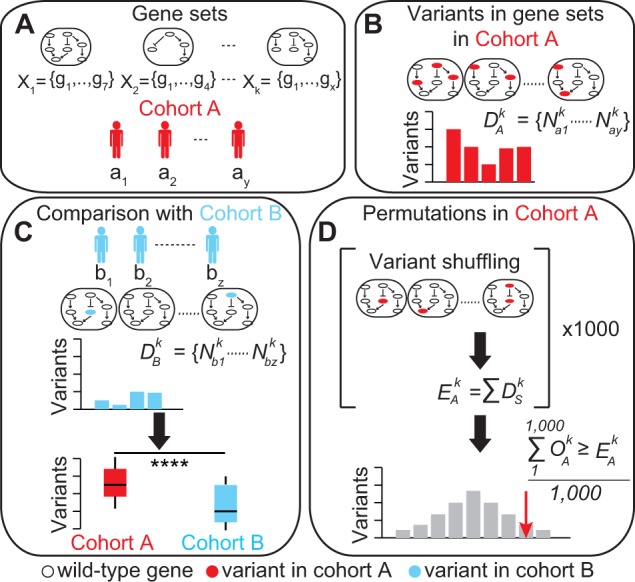
MEGA-V uses the lists of gene sets and variants in cohort A (**A**) to derive the cumulative counts of variants in each gene set (**B**) and identify the enriched gene sets using a comparison cohort B (**C**) or Monte Carlo permutations (**D**)

The purpose of MEGA-V is to identify gene sets that show a significantly higher number of variants in a cohort of interest (cohort A, Fig. 1). A gene set is defined as a group of genes *X_k_  = {g_1_,…, g_x_}* that share common features, such as biological processes from curated databases, or genes associated to the same disease (Fig. 1A). In addition to predefined gene sets, MEGA-V requires the list of variants in each gene *g* of the gene set *X_k_* for each individual *a_i_* of cohort *A = *{*a_1_,…, a_y_*} (Fig. 1A). Variants are also predefined by the user and can be damaging mutations as well as other types of genetic alterations. Once the input files are provided, the cumulative variant count $N_{a_{i}}^{k}$ within each gene set *X_k_* is computed for each individual *a_i_* in the cohort:

$$N_{a_{i}}^{k}=\sum_{g\in X_{k}}M_{a_{i},g}^{k}$$

where $M_{a_{i},g}^{k}$ is the number of variants in gene *g* of the gene set *X_k_* for individual *a_i_*. The corresponding distribution of variant counts $D_{A}^{k}$ for the gene set X_k_ (Fig. 1B) is derived as:

$$D_{A}^{k}=\left\{N_{a_{i}}^{k},\ldots ,N_{a_{y}}^{k}\right\}$$

To identify the variant gene sets in cohort A, two approaches can be applied. In the first approach, a cohort *B = {b_1_,…, b_z_*} is used to compare each distribution of variant counts $D_{A}^{k}$ to the corresponding distribution $D_{B}^{k}=\left\{N_{b}^{k},\ldots ,N_{b_{z}}^{k}\right\}$ (Fig. 1C), using Wilcoxon rank-sum test or Kolmogorov–Smirnov test according to the type of data. If multiple gene sets are tested, the resulting *P*-values are corrected for multiple testing (Benjamini and Hochberg, 1995). When the sample size of the two cohorts differs substantially, a bootstrapping procedure (random sampling with replacement) can be applied, where the larger cohort is randomly down-sampled to the size of the smaller cohort for 1000 times. At each iteration, the distributions of variants within each gene set are compared between the two cohorts and the proportion of significant enrichments (*P*-value < 0.05) over the total comparisons is calculated. In the second approach, no control cohort is used and for each gene set *X_k_* the total number of observed variants in cohort A $O_{A}^{k}$:

$$O_{A}^{k}=\sum D_{A}^{k}$$

is compared with the expected number of variants $E_{A}^{k}$:

$$E_{A}^{k}=\sum D_{S}^{k}$$

where S indicates the s-th out of 1000 Monte Carlo permutations, where the total number of variants in cohort A is randomly distributed without overlap across all genes. The empirical *P*-value for each gene set *X_k_* is measured as the fraction $O_{A}^{k}$ that is greater or equal than $E_{A}^{k}$ (Fig. 1D).

## 3 Performance assessment

To assess the performance of MEGA-V in detecting enriched gene sets, we set up a simulation study using two cohorts A and B each consisting of 100 individuals. Each individual carried 5267 mutations randomly assigned across 5267 unique genes of 186 biological pathways (Subramanian *et al.*, 2005). We simulated five conditions where one additional mutation was randomly added in one of the 186 gene sets of 20, 40, 60, 80, 100 individuals of cohort A, respectively, for a total of 930 simulations. In each simulation, we run MEGA-V, ranked the 186 gene sets according to their *P*-values, and derived the corresponding receiver operating characteristic (ROC) curve (Supplementary Data). The average ROC curves in each condition show that MEGA-V performed better as compared to randomly ranked gene sets (Supplementary Fig. S1).

## 4 Implementation

MEGA-V is implemented as a R application and is freely available on Github to be run locally or through a shiny web interface. MEGA-V requires two input files, one for the gene sets and one for the list of variants in cohort A. If cohort B is used for comparison, the associated list of variants is also required. Pre-processed gene sets of biological pathways (Subramanian *et al.*, 2005) and diseases (Cereda *et al.*, 2016) are provided. Alternatively, the user can define customised gene sets as a tab-separated file with one row per gene set specifying the gene set name and the gene symbols. The variant list is a tab separated file, with the gene symbols in the first column and the number of variants in each patient in the remaining columns. The results of the statistical analysis are summarised in a text file reporting, for each gene set, the results of the applied statistics (Wilcoxon rank-sum test, Kolmogorov–Smirnov, or Monte Carlo permutations).

## 5 Conclusions

MEGA-V provides a statistical framework to test associations between any type of perturbed biological processes and disease. For example, using MEGA-V, we have identified significantly mutated immune gene sets in individuals with multiple colorectal cancers (Cereda *et al.*, 2016).

## Supplementary Material

Supplementary Data
