# Supplementary material for: MEGA-V: detection of variant gene sets in patient cohorts
Source: Bioinformatics. 2016 Dec 21;33(8):1248–9. doi: 10.1093/bioinformatics/btw809 (PMC5408849; doi:10.1093/bioinformatics/btw809)
Supplement: Supplementary Data [file btw809_supp.doc]

**MEGA-V: detection of variant gene sets in patient cohorts**

**Supplementary Data**

To assess the performance of MEGA-V to detect enriched gene sets, we set out to perform a simulation study whereby the genetic model of the disease was set *a priori*.

We started from two cohorts A and B consisting of 100 individuals each and with every individual carrying 5,267 mutations randomly assigned across 5,267 unique genes of 186 KEGG gene sets (listed in ncomm.cereda.186.KEGG.gmt).

We then simulated five conditions where one additional mutation was randomly added in one of the 186 gene sets of 20, 40, 60, 80, 100 individuals of cohort A, respectively, for a total of 930 (186 gene sets x 5 conditions) simulations.

At each simulation, we run MEGA-V and ranked the 186 gene sets according to their p-values computed using either Wilcoxon rank-sum test or Kolmogorov Smirnov test. We then used the obtained ranked list to compute the True Positive Rate (TPR) and the False Positive Rate (FPR) at each position, defined as:

TPR = TP/(TP + FN)

and

FPR = FP/(FP + TN)

where TP were true positive predictions, *i.e.* the altered gene sets that were correctly predicted as such; FN were false negative predictions, *i.e.* the altered gene sets that were not predicted as such; and FP were false positive predictions, *i.e.* non-altered gene sets that were wrongly predicted as altered. The resulting 186 TPR and FPR values were used to derive the receivers operating characteristic (ROC) curve for that simulation.

The performance of MEGA-V in each of the five conditions was assessed by averaging the ROC curves across the 186 simulations (Supplementary Fig. 1).


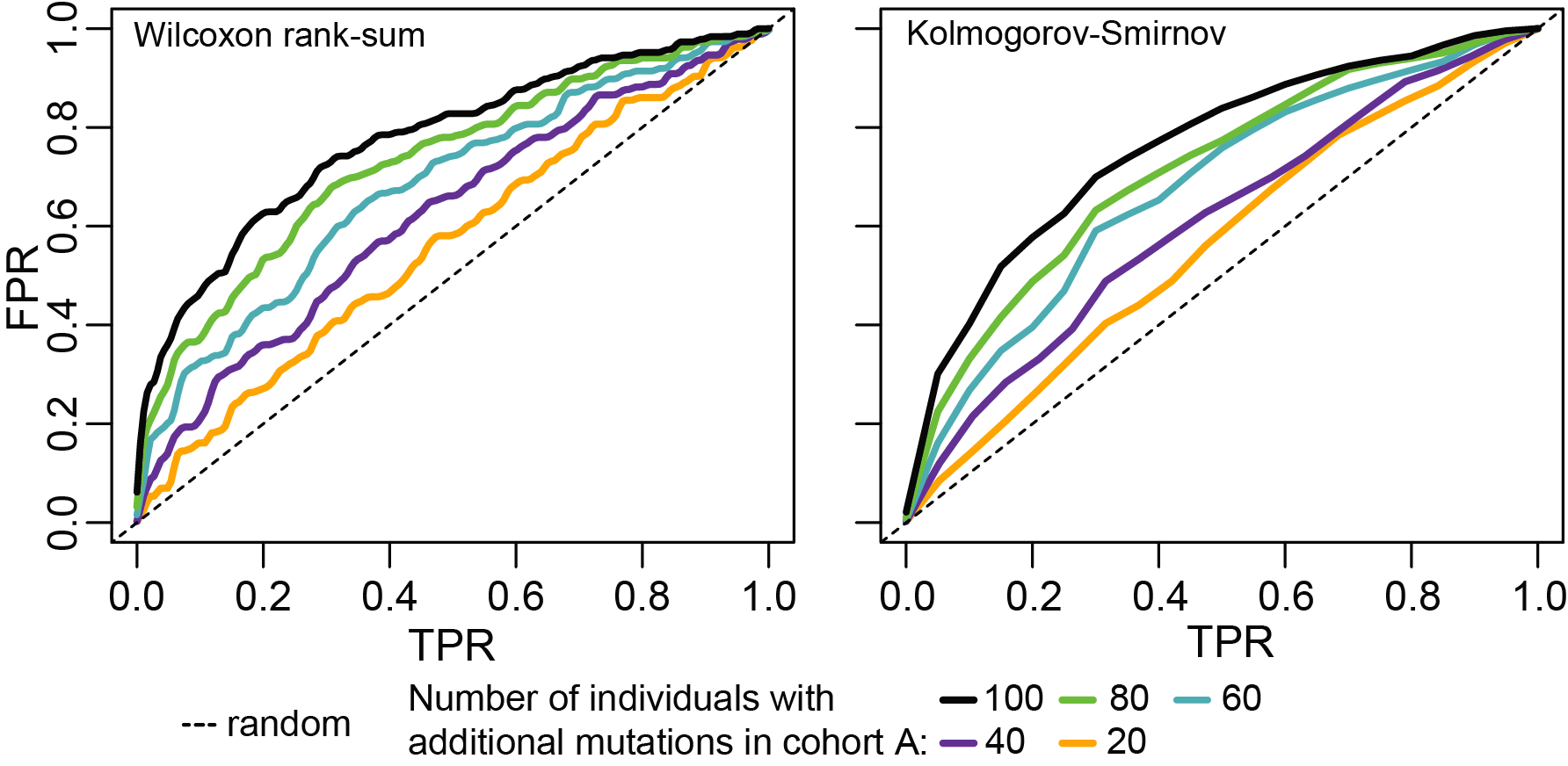


**Supplementary Fig.1 -** Assessment of MEGA-V performance using two in-silico datasets of 5,267 mutations in 5,267 unique genes of 186 KEGG gene sets. The plots represent the ROC curves for the five simulated conditions using the Wilcoxon rank-sum test (**A**) and the Kolmogorov Smirnov test (**B**) to compare the number of mutations in each gene set between the two cohorts, respectively. The dashed line represents the expected performance when ranking the 186 gene sets randomly.
